# Research priorities for psychosocial interventions in obesity care: Outcomes from a Canadian research summit

**DOI:** 10.1016/j.obpill.2026.100323

**Published:** 2026-08-19

**Authors:** S. Sockalingam, K. Park, S.L. Bacon, M. Vallis, J. Brown, I. Patton, N. Pearce, B. Agic, S.E. Cassin

**Affiliations:** a Centre for Addiction and Mental Health, Toronto, Ontario, Canada; b Department of Psychiatry, University of Toronto, Toronto, Ontario, Canada; c Bariatric Surgery Program, Toronto Western Hospital, Toronto, Ontario, Canada; d Obesity Canada, Edmonton, Alberta, Canada; e Montreal Behavioural Medicine Centre, CIUSSS-NIM, Montreal, Quebec, Canada; f Concordia University, Montreal, Quebec, Canada; g Department of Family Medicine, Dalhousie University, Halifax, Nova Scotia, Canada; h Department of Psychology, Toronto Metropolitan University, Toronto, Ontario, Canada

**Keywords:** Psychosocial interventions, Psychosocial obesity care: research priorities, Consensus development

## Abstract

**Background:**

Psychosocial and behavioural interventions are a core pillar of obesity care alongside metabolic and bariatric surgery and pharmacotherapy. However, research priorities to advance psychosocial obesity care have not yet been systematically identified in Canada.

**Objective:**

To identify national research priorities for psychosocial and behavioural interventions in obesity care from Canadian knowledge users.

**Methods:**

This was a Canadian research priority-setting consultation conducted through the virtual National Obesity Psychosocial Research Summit in September 2024. Participants included researchers, clinicians, administrators, and people with lived experience (PWLE) across Canada (N = 48). Summit attendees discussed current research priorities in psychosocial obesity care in breakout discussions followed by plenary report-backs. Facilitator notes and transcripts were analyzed using thematic analysis to generate priority areas. Participants subsequently ranked priorities in an online survey.

**Results:**

Six research priority areas were identified for psychosocial interventions in obesity care: (1) establishing systems that improve the quality and rigor of behavioural research; (2) addressing weight stigma and environmental determinants of outcomes; (3) developing consensus around research measures that evaluate the impact of behavioural treatments on outcomes other than weight (e.g., quality of life, pain reduction); (4) advancing precision-based and culturally responsive care approaches; (5) improving public, patient, and provider education regarding psychosocial care; and (6) expanding study designs and delivery modalities to enhance accessibility and scalability.

**Conclusions:**

These six priorities provide a roadmap to guide collaboration, funding, and implementation efforts to strengthen psychosocial care in obesity management.

## Graphical abstract

**Figure:**
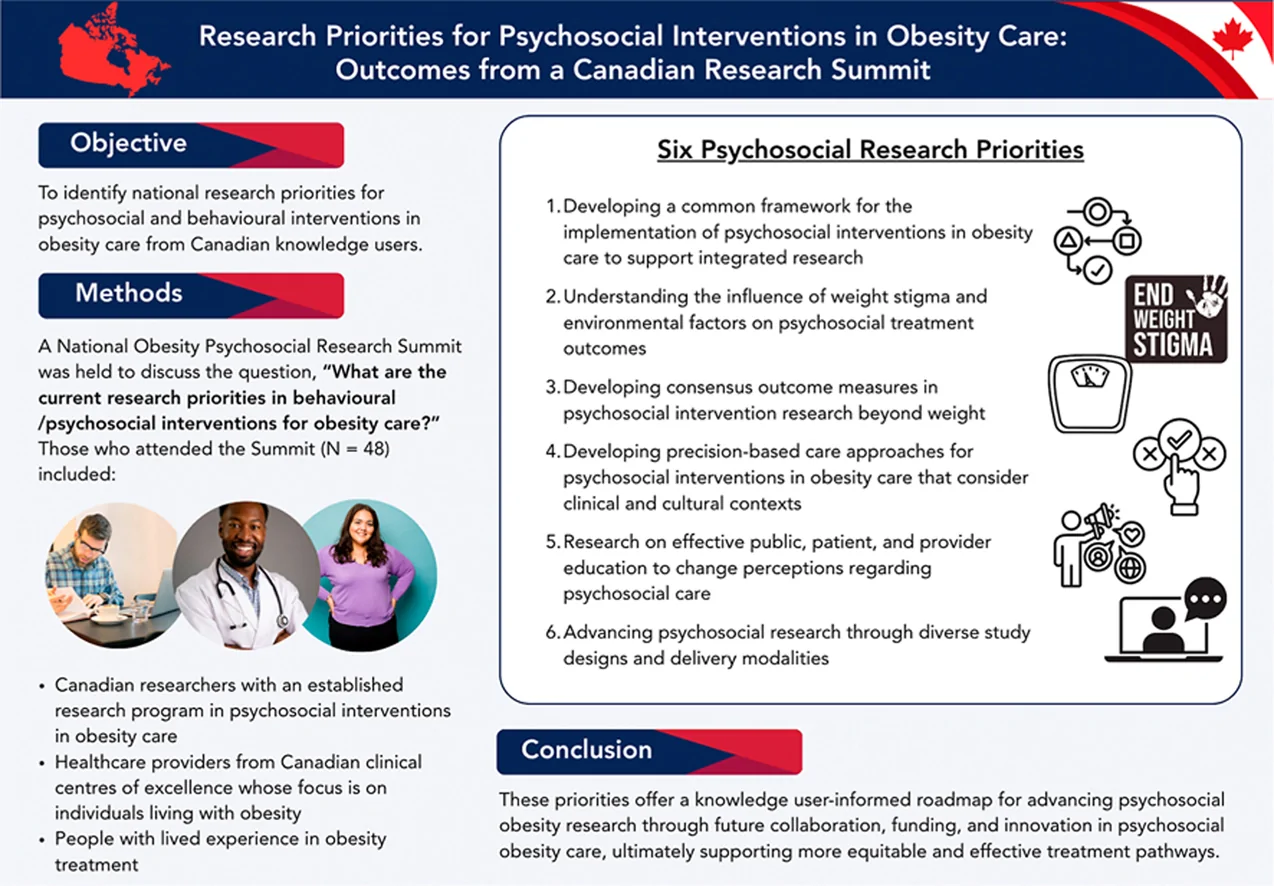

## 1 Introduction

Obesity, a chronic disease arising from a complex interplay of genetic, biological, environmental, psychological, and social factors [1], has seen major recent advancements in treatment and management approaches. The three pillars of evidence-based obesity treatment include metabolic and bariatric surgeries (MBS), pharmacotherapy, and behavioural and psychological interventions [2,3]. The number of MBS performed annually has continued to increase [4], and glucagon-like peptide-1 receptor agonists (GLP-1 RAs) and additional obesity medications facilitate weight loss and improvements in obesity-related health complications [5]. While much attention has been given to the pillars of MBS and pharmacotherapy, comprehensive obesity management also includes behavioural and psychological interventions to support sustained behaviour change [2,6,7].

Multi-component psychological and behavioural interventions are effective treatments for obesity, including reduced weight, increased treatment adherence, improved health indicators, and better quality of life [6]. Despite a paucity of psychosocial intervention studies prior to 2010 in patients undergoing MBS, recently published studies with robust methodologies [8] have reported that psychosocial interventions can improve eating pathology and psychosocial functioning [8,9]. This growing evidence underscores the importance of advancing psychosocial research that is responsive to patient needs and there is a pressing need to better understand the psychological and social factors influencing engagement, adherence, and long-term outcomes in obesity treatment.

However, a systematic review found that many psychosocial intervention studies relied on observational or uncontrolled designs, while relatively few employed randomized controlled trials with long-term follow-up [8]. Furthermore, key indicators of methodological rigor, including power calculations, blinded outcome assessment, clear behavioural change taxonomy, and treatment fidelity monitoring, were infrequently reported. These methodological limitations underscore the need for more rigorous and coordinated research in psychosocial obesity care [8,10].

The objectives of this project were to host a *National Obesity Psychosocial Research Summit* to share research currently underway in Canada, foster connections, and identify gaps and research priorities from Canadian knowledge users, i.e., researchers, clinicians, administrators, and people with lived experience (PWLE).

## 2 Methods

This was a Canadian research priority-setting consultation conducted through the virtual National Obesity Psychosocial Research Summit in September 2024. This manuscript followed the REporting guidelines for PRIority SEtting of health research (REPRISE) [11]. The intended beneficiaries of these research priorities are individuals living with obesity and healthcare providers involved in obesity care. The target audience for the priorities includes researchers, funders, policy makers, healthcare organizations, clinicians, and other stakeholders who have the capacity to implement, evaluate, or support psychosocial obesity research and care. The priorities are intended to inform research activities over the short-to medium-term and may be refined as new evidence and treatment approaches emerge.

### 2.1 Recruitment and procedure

The *National Obesity Psychosocial Research Summit* was held via Zoom on September 23rd, 2024. The Summit was overseen by the co-principal investigators of a national psychosocial obesity research initiative. The leadership team comprised a multidisciplinary group of academic researchers, healthcare providers, patient partners, and representatives from national obesity organizations, including Obesity Canada, with expertise in psychosocial obesity care, behavioural interventions, and patient-oriented research. PWLE participated throughout the planning, research, and data analysis representing meaningful engagement with co-design and co-production as collaborators and partners, as defined in patient-oriented research [12,13]. PWLE received an honorarium of $40/hr in recognition of their expertise and contributions. The leadership team was responsible for designing and overseeing the Summit, facilitating stakeholder engagement, and guiding the interpretation and refinement of the final research priorities.

Those invited to the Summit included: (a) Canadian researchers with an established research program in psychosocial interventions in obesity care; (b) healthcare providers from Canadian clinical centres of excellence whose focus is on individuals living with obesity; and (c) PWLE in obesity treatment identified through existing regional and national networks (i.e., Obesity Canada). Invitees were invited via email and asked to forward the invitation to others who may be interested in contributing to the discussion of psychosocial interventions in obesity care (i.e., snowball sampling). We aimed to have 40–50 participants attend the Summit to balance representation from diverse stakeholder groups and geographic regions across Canada with the need to facilitate meaningful breakout discussions and provide sufficient time for each group to share and discuss priorities during the plenary session.

To begin the Summit, researchers with established psychosocial intervention research programs presented on current work being conducted across Canada. Participants then joined breakout rooms to discuss the question, “*What are the current research priorities in behavioural/psychosocial interventions for obesity care?”* and returned for a single share back (see supplementary materials for further details). The Summit was audio recorded and facilitators took notes within six breakout rooms. Following the Summit, registrants were asked to complete a brief online quantitative survey to rank the identified research priorities in order of importance.

### 2.2 Data analyses

During the Summit, facilitators in each breakout room documented key discussion points and proposed research priorities using a standardized tracking sheet. Each group then presented its priorities during a plenary report-back, during which an administrative host with expertise in group facilitation, who had not participated in the breakout discussions, documented the priorities and facilitated discussion among all attendees. Through these facilitated discussions, attendees identified nine preliminary research priorities considered most important for advancing behavioural and psychosocial interventions in obesity care.

Following the Summit, one member of the research team reviewed all facilitator notes and the audio recording to verify, organize, refine, and consolidate the identified priorities. Summit co-leads, including the principal investigators, subsequently met on several occasions to review the preliminary priorities. During these meetings, the preliminary synthesis was presented and discussed, and decisions regarding the organization and consolidation of overlapping priorities were reached through iterative group consensus. Using an inductive thematic approach, the research team grouped related priorities into broader themes that emerged from participant discussions rather than from a predefined framework. Through this iterative process, the preliminary priorities were refined and organized into six final research priority themes by merging conceptually related priorities. Each theme was accompanied by examples of potential research activities that illustrate how the priorities could be operationalized in future studies.

After the research priorities were finalized, Summit participants were invited to complete an online survey ranking each priority from #1 (most important) to #6 (least important). The final ordering of research priorities were based on mean rank. If two priorities had the same mean, ranking was decided by the lower standard deviation. Kendall's W was calculated to identify rank correlation. The post-Summit ranking survey also included an open-text field for additional comments on the identified priorities.

## 3 Results

### 3.1 Participant characteristics

A total of 48 participants attended the Summit. Twenty-eight participants (58.3%) subsequently completed the follow-up survey. Participant descriptive statistics appear in Table 1.

**Table 1:** Characteristics of Participants who Attended the Summit (*N* = 48).

Characteristic	*n* (%)^a^
**Role**	
Administrator (e.g., program manager)	5 (10.4%)
Nurse or Nurse Practitioner	3 (6.3%)
Researcher	18 (37.5%)
Dietitian	6 (12.5%)
Person with Lived Experience/Family Member	9 (18.8%)
Family Physician	1 (2.1%)
Specialist Physician	2 (4.2%)
Psychologist	8 (16.7%)
Pharmacist	3 (6.3%)
Social Worker	1 (2.1%)
Student	7 (14.6%)
Other	1 (2.1%)
Prefer not to answer	0 (0%)
Missing	0 (0%)

**Region of Canada**	
Atlantic	6 (12.5%)
Quebec	3 (6.3%)
Ontario	21 (43.8%)
Prairies	9 (18.8%)
British Columbia	0 (0%)
Territories	0 (0%)
Canada (province/territory unspecified)	2 (4.2%)
Missing	6 (12.5%)
Outside of Canada	1 (2.1%)

**Gender**	
Cis-Woman	35 (72.9%)
Cis-Man	10 (20.8%)
Non-binary	1 (2.1%)
Missing	1 (2.1%)

**Ethnicity**^b^	
Asian - East	2 (4.2%)
Asian - Southeast	1 (2.1%)
Black	2 (4.2%)
Middle Eastern	1 (2.1%)
White	36 (75%)
Mixed heritage	2 (4.2%)
Missing	8 (16.7%)

a Percentages do not total 100% because participants could select more than one option.

b Additional ethnicity options were offered but were not selected.

### 3.2 Themes (Research priorities)

**Fig. 1:**
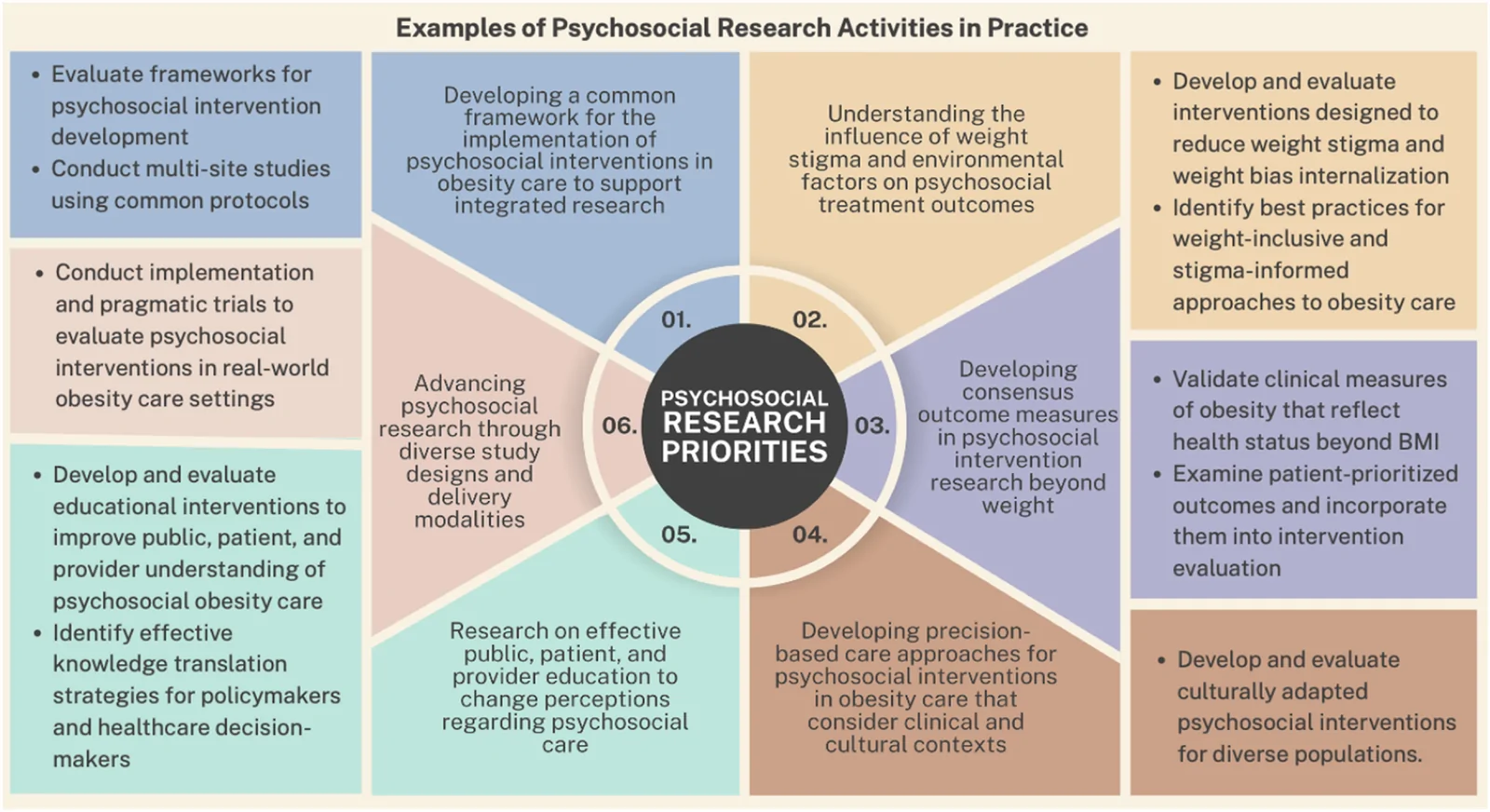
Research priorities and example research activities for psychosocial obesity care.

Themes are presented in order of mean priority (see supplementary materials for the mean ranking of each priority and the frequency of each rank). While the agreement among participants' rankings reached statistical significance, the overall strength or level of consensus was relatively low, Kendall's *W* = 0.22, χ^2^ (5) = 31.0, *p* < 0.001. Examples of research questions and methodological approaches relevant to each priority are presented in Fig. 1.

#### 3.2.1 Theme 1. Developing a common framework for the implementation of psychosocial interventions in obesity care to support integrated research

Summit participants highlighted concerns about the methodological rigor of many obesity-related behavioural intervention trials, noting that studies often lacked well-defined processes for intervention development, piloting, and evaluation [14]. Unlike pharmacological research, which relies on well-established frameworks [15], behavioural intervention research has been criticized for limited uptake of evaluation frameworks [14], such as the ORBIT framework (Obesity-Related Behavioral Intervention Trials) [16] or the Medical Research Council's complex intervention guidance [17]. Limited funding opportunities for early stage intervention development further restricts the use of these approaches.

A shared implementation framework would support more integrated and coordinated approaches to psychosocial care by providing a common structure for intervention development, evaluation, and reporting. This would facilitate collaboration across research groups, improve comparability of findings, reduce duplication of effort, and accelerate the translation of effective interventions into clinical practice. A coherent framework would ultimately enhance scalability and reduce fragmentation in psychosocial obesity care. Similar to the role of CONSORT [18] in improving the consistency of randomized controlled trials, a shared implementation framework could be promoted through endorsement by funding agencies, journals, and professional organizations, thereby encouraging broader adoption and improving comparability across studies.

#### 3.2.2 Theme 2. Understanding the influence of weight stigma and environmental factors on psychosocial treatment outcomes

Weight stigma, both societal and internalised, can undermine treatment engagement and reduce the effectiveness of psychosocial interventions in obesity care [19]. Research is needed to clarify how stigma and structural barriers shape treatment processes and outcomes, and to develop interventions that directly address these influences. Importantly, stigma- and equity-informed principles should also be integrated into the design and delivery of all psychosocial interventions, rather than limited to stand-alone or targeted programs. Advancing this priority will support the creation of psychosocial approaches that promote self-compassion, reduce stigma-related barriers, and enhance equitable access to care.

#### 3.2.3 Theme 3. Developing consensus outcome measures in psychosocial intervention research beyond weight

While obesity is understood to be a complex chronic disease and the limitations of simplistic BMI measurements are well established [20], research and clinical settings continue to focus on weight/BMI as primary outcome measures. There is an urgent need for more holistic understanding of health and treatment effectiveness, incorporating factors such as quality of life, daily functioning, mental health, and pain. The International Consortium for Health Outcomes Measurement (ICHOM) has developed a recommended set of standardised outcomes for adult obesity care [21]. Implementing these measures (and other standardised psychosocial outcomes) in research can facilitate multi-site comparisons and advance the field by promoting more consistent and comprehensive reporting of psychosocial outcomes. Relatedly, when defining obesity in research and clinical practice, there is a need to develop clinical measures of obesity that reflect overall health, rather than just body size, and with standardised assessment frameworks that still allow outcomes to be prioritised according to individual patient goals [5,22].

#### 3.2.4 Theme 4. Developing precision-based care approaches for psychosocial interventions in obesity care that consider clinical and cultural contexts

Psychosocial intervention research in obesity care should identify key predictors that determine *what works, for whom, and when* in obesity care interventions. Patients, researchers, and clinicians have identified a need for more precise approaches that are tailored to individual needs and circumstances [23]. Obesity care intervention research should explore how interventions can be more inclusive and culturally adaptive, given the impact of obesity on diverse populations and high-risk groups. Factors such as diverse nutritional preferences, cultural traditions, and values will impact psychosocial intervention engagement. Furthermore, there is a need to use large data sets that include qualitative and quantitative data and leverage artificial intelligence (AI) to increase precision-based approaches to psychosocial intervention selection.

#### 3.2.5 Theme 5. Research on effective public, patient, and provider education to change perceptions regarding psychosocial care

Misconceptions about the role and value of psychosocial interventions in obesity care remain widespread [14]. Summit participants identified the need for effective educational and knowledge translation strategies that enhance awareness of how psychosocial interventions support health outcomes, including adherence to medical treatments, behaviour change, and long-term maintenance. Relatedly, it is a priority to better understand how decision-makers and funding bodies conceptualise psychosocial care, and how researchers can align communication strategies with these perspectives to support resource allocation.

#### 3.2.6 Theme 6. Advancing psychosocial research through diverse study designs and delivery modalities

Summit participants concurred that future psychosocial research should utilise varied study designs when evaluating the effectiveness of psychosocial treatment options in obesity care. This includes longitudinal designs and implementation-focused studies that clarify how interventions can be delivered and scaled in real-world settings. Additionally, this priority emphasises the need to explore diverse delivery modalities for interventions, including technology-enabled care, such as app-based (with or without AI) approaches, peer-supported models, virtual interventions, and traditional face-to-face methods. Prioritising these diverse approaches will enhance accessibility and scalability and strengthen the overall impact of psychosocial care in obesity treatment.

## 4 Discussion

This Summit represents one of the first coordinated national efforts in Canada to identify research priorities specifically focused on psychosocial and behavioural interventions in obesity care. The six priorities reflect a shared recognition that psychosocial approaches are essential across all obesity treatments and that advancing this field requires more consistent, patient-centred, and methodologically rigorous research. Establishing these priorities provides a roadmap for strengthening the evidence base in several key areas, including the use of standardised frameworks for intervention design [14,16], addressing weight stigma and structural barriers that undermine engagement [1,7,19], and improving the consistency of psychosocial outcome measurement [21,22]. The identification of these priorities also underscores the importance of precision-based and culturally responsive approaches [23], as well as the need to broaden education about the value of psychosocial care [14]. Finally, participants highlighted the potential of diverse study designs and delivery modalities to improve scalability and access.

This research priority-setting consultation has limitations. Participants were predominantly White, cisgender women and represented a convenience sample of individuals already engaged in obesity research and clinical care, which may have influenced the priorities identified. Nevertheless, these findings provide an important starting point for future priority-setting efforts that intentionally engage more diverse stakeholders to refine and expand the psychosocial obesity research agenda. Additionally, although participants had opportunities to discuss the preliminary priorities during the Summit and provide additional feedback through the post-Summit survey, formal member checking of the final synthesized themes was not undertaken.

## 5 Conclusion

This Summit identified six research themes to guide the future development, evaluation, and implementation of psychosocial interventions in obesity care. Collectively, these priorities offer a knowledge user-informed roadmap for advancing psychosocial obesity research through future collaboration, funding, and innovation in psychosocial obesity care, ultimately supporting more equitable and effective treatment pathways.

## 6 Key clinical takeaways

• Despite advances in surgical and pharmacological obesity treatment, psychosocial care remains under-integrated in routine care despite its important role in supporting behaviour change and long-term outcomes.
• Advancing psychosocial intervention trials requires attention to weight stigma and structural barriers that undermine treatment engagement, and the development of precision-based and culturally responsive approaches.
• Future psychosocial obesity care should prioritize outcome measures beyond weight and support accessible, scalable models of care through improved education, diverse delivery modalities, and rigorous evaluation.

## Author disclosures

The authors declared no conflict of interest.

## Ethical review

Ethics approval was not obtained as the Summit was conducted as a stakeholder engagement and knowledge-sharing activity. In accordance with Article 2.5 of the Tri-Council Policy Statement: Ethical Conduct for Research Involving Humans (TCPS 2), activities undertaken for quality assurance, quality improvement, program evaluation, or other non-research purposes do not constitute research requiring research ethics board review. All participants were informed prior to data collection that their Summit participation was voluntary, and findings would be reported only in aggregate, with no identifying information attributed to individual participants. In addition, data collection did not meet quality improvement review by our institution.

## CRediT authorship contribution statement

SS: Conceptualization, Methodology, Formal analysis, Writing – original draft, Supervision, Funding acquisition; KP: Formal analysis, Investigation, Data curation, Writing – original draft, Visualization, Project administration; SLB: Conceptualization; MV: Conceptualization; IP: Resources, Project administration; NP: Resources, Project administration; BA: Conceptualization, Visualization, Funding acquisition; SEC: Conceptualization, Methodology, Formal analysis, Writing – original draft, Supervision, Funding acquisition. Additionally, all authors reviewed, edited, and approved the final submission.

## Artificial intelligence (AI)

AI was used to assist with grammar editing and reference formatting. All content was reviewed and approved by the authors.

## Funding

The CIHR Planning and Dissemination Grants - (#505740).

## Appendix A Supplementary data

**Supplementary materials:** Completed Reporting Guideline for Priority Setting of Health Research (REPRISE) checklist; National Obesity Research Summit agenda; facilitator probing questions for breakout discussions; mean ranking table for research priorities; and graphs depicting the distribution of rankings assigned to each research priority by participants.
