# Supplementary material for: Research priorities for psychosocial interventions in obesity care: Outcomes from a Canadian research summit
Source: Obes Pillars. 2026 Aug 19;19:100323. doi: 10.1016/j.obpill.2026.100323 (PMC13524578; doi:10.1016/j.obpill.2026.100323)
Supplement: Supplementary file 1 [file mmc1.docx]

**Supplementary Materials**

**Supplementary Table S1. Reporting guideline for health research priority setting with stakeholders (REPRISE)**

| **No** | **Item** | **Descriptor and/or examples** | **Page** |
| --- | --- | --- | --- |
| **A** | **Context and Scope** |  |  |
| 1 | Define geographical scope | Global, regional, national, city, local area, institutional/organizational level, health service | National (Canada)  Page 3 |
| 2 | Define health area, field, focus | Disease or condition specific, interventions, healthcare delivery, health system | Obesity  Page 3 |
| 3 | Define the intended beneficiaries | This may include the general population or a specific population based on demographic (age, gender), clinical (disease, condition), or other characteristics who may benefit from the research | Page 4 |
| 4 | Define the target audience of the priorities | Policy makers, funders, researchers, industry or others who have the potential to implement the priorities identified | Page 4 |
| 5 | Identify the research area | Public health, health services research, clinical research, basic science | Page 4 |
| 6 | Identify the type of research questions | Etiology, diagnosis, prevention, treatment (interventions), prognosis, health services, psychosocial, behavioral and social science, economic evaluation, implementation; this may not be pre-defined | Page 4 |
| 7 | Define the time frame | Interim, short-term, long-term priorities, plans to revise and update | Page 4 |
| **B** | **Governance and team** |  |  |
| 8 | Describe the selection and structure of the leadership and  management team | Those responsible for initiating, developing, and guiding the process  for priority setting, and examples of structures include; Steering  Committee, Advisory Group, Technical Experts | Page 4 |
| 9 | Describe the characteristics of the team | Stakeholder group or role, institutional affiliations, country or region,  demographics (e.g. age sex), discipline, experience, expertise | Page 4 |
| 10 | Describe any training or experience relevant to conducting  priority setting | Consultants or advisors, members with experience or skills relevant to the conducting priority-setting e.g. qualitative methods, surveys,  facilitation | Page 5 |
| **C** | **Framework for priority setting** |  |  |
| 11 | State the framework used (if any) | James Lind Alliance, COHRED, CHNRI, Dialogue Model, no framework (general research priority setting) | No framework  Page 6 |
| **D** | **Stakeholders or participants** |  |  |
| 12 | Define the inclusion criteria for stakeholders involved in priority-setting | Patients, caregivers, general community, health professionals, researchers, policy makers, non-governmental organizations, government, industry; specific groups including vulnerable and marginalized populations | Page 5 |
| 13 | State the strategy or method for identifying and engaging stakeholders | Partnership with organizations, social media, recruitment through hospitals | Page 5 |
| 14 | Indicate the number of participants and/or organizations involved | Number of individuals and organizations, include number by stakeholder group | Page 6 |
| 15 | Describe the characteristics of stakeholders | Stakeholder group, demographic characteristics, areas of interest and expertise, discipline, affiliations | Page 6 & Table 1 |
| 16 | State if reimbursement for participation was provided | Cash, vouchers, certificates, acknowledgement; what purpose e.g. travel, accommodation, honorarium | Page 4  (Note: Only PWLE were given honorariums) |
| **E** | **Identification and collection of research priorities** |  |  |
| 17 | Describe methods for collecting initial priorities | Methods e.g. Delphi survey, surveys, nominal group technique, interviews, focus groups, meetings, workshops; prioritization e.g. voting, ranking; mode e.g. face-to-face, online; may be informed by evidence e.g. systematic reviews, reviews of guidelines/other documents, health technology assessment | Page 5 |
| 18 | Describe methods for collating and categorizing priorities | Taxonomy or other framework used to organize, summarise, and aggregate topics or questions | Page 6 |
| 19 | Describe methods and reasons for modifying (removing, adding, reframing) priorities | Based on scope, clarity, definition, duplication, other criteria | Page 6 |
| 20 | Describe methods for refining or translating priorities into research topics or questions | Reviewed by Steering Committee or project team | Page 6 |
| 21 | Describe methods for checking whether research questions or topics have been answered | Systematic reviews, evidence mapping, consultation with experts | Overview of existing research was provided by experts in the field and informed group discussions – Page 5 |
| 22 | Describe number of research questions or topics | Number of priorities at each stage of the process | Pages 5 & 6 |
| **F** | **Prioritization of research topics/questions** |  |  |
| 23 | Describe methods and criteria for prioritizing research topics or questions | Methods e.g. Delphi survey, surveys, nominal group technique, interviews, focus groups, meetings, workshops; Prioritization e.g. voting, ranking; Mode e.g. face-to-face, online; Criteria e.g. need, feasibility, novelty, equity | Page 6 |
| 24 | State the method or threshold for excluding research topics/questions | Thresholds for ranking scores, proportions, votes; other criteria | Page 6 |
| **G** | **Output** |  |  |
| 25 | State the approach to formulating the research priorities | Area, topic, questions, PICO (population, intervention, comparator, outcome) | Page 5 and Figure 1 |
| **H** | **Evaluation and feedback** |  |  |
| 26 | Describe how the process of prioritization was evaluated | Survey, workshop | Page 7 |
| 27 | Describe how priorities were fed back to stakeholders and/or to the public; and how feedback (if received) was addressed and integrated | Public meetings or workshop, newsletters, website, email, online presentations | Stakeholders (researchers, clinicians, administrators, and PWLE) were present throughout the process  Dissemination includes conference presentations and planned stakeholder newsletters |
| **I** | **Implementation** |  |  |
| 28 | Outline the strategy or action plans for implementing priorities | Communication with target audience, via policies and funding | Page 12 |
| 29 | Describe plans, strategies, or suggestions to evaluate impact | Integration in decision-making, funding allocation, review of relevant documents | N/A  - No formal strategy to evaluate the impact of the identified priorities has been established  - Impact may be reflected in future funding priorities, research collaborations, and the design of psychosocial obesity research |
| **J** | **Funding and conflict of interest** |  |  |
| 30 | State sources of funding | Name sources of funding for the priority-setting exercise; if relevant include the budget and/or cost | Title Page |
| 31 | Declare any conflicts or competing interests | State any conflicts of interest that may be at an individual level and/or at a contextual level (e.g. political issues, controversies) that may affect the process, output or implementation. | Title Page (none to disclose) |

**Supplementary Figure S1: National Obesity Research Summit Agenda**

**Supplementary Material S1: Facilitator Probing Questions for Breakout Discussions**

- Key Question: What are the current research priorities in behavioural/psychosocial interventions for obesity care?

*Probing questions:*

- After hearing our symposium, are there areas where we should focus next? What research would help patients living with obesity?
- What research priorities are not on the list but should be there?
- What will be facilitators to addressing these gaps?
- What do we need to be successful in advancing the research in these areas?

**Supplementary Table S2.** Ranking of Research Priorities (*n* = 28)

| **Rank** | **Research Priority** | ***M*** | ***SD*** |
| --- | --- | --- | --- |
| 1 | Developing a common framework for the implementation of psychosocial interventions in obesity care to support integrated research | 2.86 | 1.24 |
| 2 | Understanding the influence of weight stigma and environmental factors on psychosocial treatment outcomes | 2.86 | 1.67 |
| 3 | Developing consensus outcome measures in psychosocial intervention research beyond weight | 3.11 | 1.57 |
| 4 | Developing precision-based care approaches for psychosocial interventions in obesity care that consider clinical and cultural contexts | 3.14 | 1.58 |
| 5 | Research on effective public, patient and provider education to change perceptions regarding psychosocial care | 3.93 | 1.56 |
| 6 | Advancing psychosocial research through diverse study designs and delivery modalities | 5.11 | 1.55 |

Note: Lower mean scores represent higher research priority ranking

**Supplementary Figure S2.** Frequency of Research Priorities (*n* = 28)

Developing Common Framework

|  | Frequency |
| --- | --- |
| Ranked 1 | 5 |
| Ranked 2 | 6 |
| Ranked 3 | 7 |
| Ranked 4 | 8 |
| Ranked 5 | 2 |
| Ranked 6 | 0 |

Influence of Weight Stigma

|  | Frequency |
| --- | --- |
| Ranked 1 | 8 |
| Ranked 2 | 7 |
| Ranked 3 | 1 |
| Ranked 4 | 7 |
| Ranked 5 | 3 |
| Ranked 6 | 2 |

Outcome Measures

|  | Frequency |
| --- | --- |
| Ranked 1 | 5 |
| Ranked 2 | 7 |
| Ranked 3 | 4 |
| Ranked 4 | 6 |
| Ranked 5 | 4 |
| Ranked 6 | 2 |

Precision-Based Care

|  | Frequency |
| --- | --- |
| Ranked 1 | 6 |
| Ranked 2 | 2 |
| Ranked 3 | 11 |
| Ranked 4 | 4 |
| Ranked 5 | 11 |
| Ranked 6 | 3 |

Patient/Provider Education

|  | Frequency |
| --- | --- |
| Ranked 1 | 3 |
| Ranked 2 | 3 |
| Ranked 3 | 4 |
| Ranked 4 | 4 |
| Ranked 5 | 11 |
| Ranked 6 | 3 |

Diverse Delivery Modalities

|  | Frequency |
| --- | --- |
| Ranked 1 | 1 |
| Ranked 2 | 3 |
| Ranked 3 | 1 |
| Ranked 4 | 0 |
| Ranked 5 | 5 |
| Ranked 6 | 18 |
